# Transcriptome Analysis Reveals the Molecular Response to Salinity Challenge in Larvae of the Giant Freshwater Prawn *Macrobrachium rosenbergii*

**DOI:** 10.3389/fphys.2022.885035

**Published:** 2022-04-29

**Authors:** Yakun Wang, Jie Wei, Kunhao Hong, Nan Zhou, Xiaoli Liu, Xiaoyou Hong, Wei Li, Jian Zhao, Chen Chen, Liang Wu, Lingyun Yu, Xinping Zhu

**Affiliations:** ^1^ Key Laboratory of Tropical and Subtropical Fishery Resource Application and Cultivation, Ministry of Agriculture and Rural Affairs, Pearl River Fisheries Research Institute, Chinese Academy of Fishery Sciences, Guangzhou, China; ^2^ College of Fisheries and Life Science, Shanghai Ocean University, Shanghai, China; ^3^ School of Fishery, Zhejiang Ocean University, Zhoushan, China; ^4^ Sisal and Sisal Products Quality Supervision, Inspection and Testing Center, Ministry of Agriculture and Rural Affairs, Zhanjiang, China

**Keywords:** salinity challenge, molecular response, transcriptome analysis, RNAseq, Macrobrachium rosenbergii

## Abstract

Salinity is a crucial factor influencing the growth, development, immunity, and reproduction of aquatic organisms; however, little is known about the molecular mechanism of the response to salinity challenge in larvae of the giant freshwater prawn *Macrobrachium rosenbergii*. Herein, larvae cultured in three treatment groups with salinities of 10, 13, and 16‰ (S10, S13, and S16) were collected, and then transcriptome analysis was conducted by RNA-seq. A total of 6,473, 3,830 and 3,584 differentially expressed genes (DEGs) were identified in the S10 vs. S13 comparison, S10 vs. S16 comparison and S13 vs. S16 comparison, respectively. These genes are involved in osmoregulation, energy metabolism, molting, and the immune response. qPCR analysis was used to detect the expression patterns of 16 DEGs to verify the accuracy of the transcriptome data. Protein–protein interaction (PPI) analysis for DEGs and microsatellite marker screening were also conducted to reveal the molecular mechanism of salinity regulation. Together, our results will provide insight into the molecular genetic basis of adaptation to salinity challenge for larvae of *M. rosenbergii*.

## Introduction

Aquatic organisms usually suffer from fluctuating water environment effectors, of which salinity is one of the key factors affecting growth, development, metabolism, immunity, and reproduction (Yen and Bart, 2008; Zhang et al., 2016; Huang et al., 2019a; Wen et al., 2021; Yi et al., 2021). In general, salinity affects aquatic animals’ metabolism by influencing the osmotic pressure of tissues. Organisms usually absorb more water from the environment and secrete ions into the environment under low-salinity conditions, whereas under high-salinity conditions, they release more water into the environment and absorb more ions from the environment through active transportation. In this way, osmoregulation is well regulated (Wilson et al., 2002; Perry et al., 2006; Whittamore, 2012). Therefore, organisms can respond to salinity changes in different ways to maintain internal environmental homeostasis.

Due to their direct exposure to environmental salinity, aquatic animals typically suffer from several kinds of metabolic pressures, including osmoregulation, oxidative stress, lipid metabolism, and gut microbes (Zhang et al., 2016; Giffard-Mena et al., 2020; Dawood et al., 2021; Liu et al., 2021). Long-term salt exposure will damage the growth, osmotic condition, and immune response of aquatic organisms to some extent (Dawood et al., 2021). Variation in salinity may also cause physiological stress through changes in plasma hormones, energy metabolism and electrolyte balance, and these stressors can induce oxidative damage (Choi et al., 2008). However, salinity exposure is not always harmful. Previous studies have shown that a certain salinity is necessary and beneficial for growth and development; for example, female *Macrobrachium rosenbergii* cultured under salinities of 0 and 6 gL^−1^ produced more eggs, and the hatchling rate was higher than that under 12 gL^−1^ (Yen and Bart, 2008). Fry of the freshwater fish *Ompok pabda* reared at 2.5‰ salinity mostly survived (Alam et al., 2020). Short-term salinity exposure will help to improve the immunity of the pufferfish *Takifugu fasciatus* (Wen et al., 2021). To date, the molecular mechanisms underlying the response to salinity challenge in aquatic animals are poorly understood.

Transcriptome analysis, one of the methods available for investigating the response to salinity stress, has been applied to many aquatic animal species, such as the marbled sole *Pseudopleuronectes yokohamae* (Cui et al., 2019), turbot *Scophthalmus maximus* (Liu et al., 2021), Pacific white shrimp *Litopenaeus vannamei* (Hu et al., 2015), and clam *Cyclina sinensis* (Ni et al., 2021). However, to the best of our knowledge, research is still lacking regarding the transcriptome data of larvae of the giant freshwater prawn *M. rosenbergii* under salinity stress.

The giant freshwater prawn *M. rosenbergii* is inarguably the most economically important freshwater prawn cultured in Southeast Asia and China because of its high protein content and high-quality flesh (New, 2005; Wei et al., 2021). Currently, the worldwide farmed production of *M. rosenbergii* has increased from 101,509 tons in 1999 to 273,737 tons in 2019 (FAO, 2021). Previous reports have revealed the effects of salinity on growth, development, metamorphosis, and reproduction (Yen and Bart, 2008; Chand et al., 2015; Wei et al., 2021); however, little information is reported about the molecular mechanism by which salinity influences larval development. Larvae rearing is the critical event for successful seed production under artificial breeding conditions because the hatching and metamorphosis of larvae require a water environment with a certain salinity (Cheng et al., 2003; Yen and Bart, 2008). Therefore, this study aimed to clarify the molecular mechanism of this process, which will provide key information for seed breeding and contribute to the sustainable development of the *M. rosenbergii* aquaculture industry.

## Materials and Methods

### Ethics Statement

All the experimental procedures involving the larvae in this experiment were approved by the Experimental Animal Care and Ethics Committee of the Pearl River Fisheries Research Institute, Chinese Academy of Fishery Sciences.

### Experimental Design and Sample Collection

According to our recent study (Wei et al., 2021), the larvae of *M. rosenbergii* newly hatched in the same incubation pond were from Foshan Sanshui Baijin Seedling Co., Ltd. These larvae (average body weight of 0.62 ± 0.01 mg and average body length of 1.20 ± 0.02 mm) were randomly divided into three groups with salinities of 10‰ (S10), 13‰ (S13) and 16‰ (S16). Each group was replicated three times separately, and each experimental barrel had a capacity of 700 L and contained approximately 20,000 individuals. Our experiment lasted for 23 days, during which the larvae were fed fairy shrimp (*Artemia salina*) daily and waste was removed. Experimental water with the same salinity created by mixing seawater with an original salinity of 20‰ with freshwater after disinfection and full aeration was added to maintain the original water level as that in the original barrel every 3 days. The water dissolved oxygen and pH ranged from 7.25 to 8.66 mg/L and from 8.15 to 8.27, respectively, and the temperature was 30 ± 0.5°C.

Each 1.5 ml EP cryogenic vial containing approximately ten larvae collected from each experimental barrel was considered one sample, and the 9 samples from all three groups (S10, S13 and S16) were immediately frozen in liquid nitrogen and stored at −80° for further analysis.

### RNA Extrication, Library Construction and Sequencing

The total RNA of larvae was extracted using TRIzol Reagent (Invitrogen, Waltham, MA, United States) according to the manufacturer’s protocol. RNA purity and concentration were measured with a NanoDrop One UV–Vis Spectrophotometer (Thermo Fisher Scientific, Wilmington, DE, United States). RNA integrity was assessed by 1% agarose gel electrophoresis. Three micrograms of RNA per sample was used as input material for RNA sample preparation. In total, nine sequencing libraries were constructed with the NEBNext^®^ UltraTM RNA Library Prep Kit for Illumina^®^ (NEB, Beijing, China) following the manufacturer’s instructions. The library fragments were purified with a QiaQuick PCR Extraction Kit (Qiagen, Venlo, Netherlands) prior to sequencing and then sequenced using an Illumina HiSeq2500 instrument at Gene Denovo Biotechnology Co. (Guangzhou, China).

### Quality Control and Transcriptome Assembly

To obtain high-quality reads for further analysis, clean data were generated by removing reads containing adapter or poly-N sequences and low-quality reads from the raw data with fastp 0.18.0 software (Chen et al., 2018). At the same time, Q30 and GC content of the clean data were calculated. Then, high-quality reads were reconstructed and assembled into unigenes by Trinity v2.8.3 with default parameters for *de novo* transcriptome assembly (Cabau et al., 2016). The integrity of transcriptome assembly was assessed with Benchmarking Universal Single-Copy Orthologs (BUSCO) (http://busco.ezlab.org). Finally, the National Center for Biotechnology Information nonredundant (Nr, ftp://ftp.ncbi.nih.gov/blast/db/), Swiss-Protein (https://www.uniprot.org/), Kyoto Encyclopedia of Genes and Genomes (KEGG, https://www.genome.jp/kegg/), and Gene Ontology (GO, http://geneontology.org/) databases were used to obtain feature and annotation information for all assembled unigenes.

### Analysis of Differentially Expressed Genes

The fragments per kilobase of transcript per million mapped reads (FPKM) values were used to estimate gene expression levels using StringTie software (Pertea et al., 2016). The analysis of differentially expressed genes (DEGs) among the S10 group, S13 group and S16 group was performed by DESeq 2 (Love et al., 2014) with a threshold false discovery rate (FDR) of <0.05 and absolute fold change of ≥2 (log_2_^|FC|^ ≥ 1). Log _2_^(FC)^ > 0 indicates upregulation, and log _2_^(FC)^ < 0 indicates downregulation.

### Gene Ontology Enrichment and Kyoto Encyclopedia of Genes and Genomes Pathway Analysis

To obtain more information about the functions of DEGs, the GOseq R package (Young et al., 2010) and KEGG Orthology database (Ogata et al., 1999) were used to perform Gene Ontology (GO) annotation and Kyoto Encyclopedia of Genes and Genomes (KEGG) analysis. GO terms and KEGG pathways with corrected *p* values < 0.05 were identified as significantly enriched.

### Trend Analysis

To understand the expression patterns of DEGs, trend analysis was used to cluster genes with similar expression patterns in multiple samples. Clustering of the DEGs was performed by using Short Time-series Expression Miner (STEM) software (Ernst and Bar-Joseph, 2006), and clustered profiles were considered significant when *p* values were ≤0.05.

### Transcription Factor Analysis

Transcription factors (TFs) are crucial regulators of gene expression in many organisms. To obtain more information on the gene regulation of the DEGs, the predicted protein sequences were aligned to the corresponding Animal TF Database by the hmmsearch program in HMMER v3.0 (Finn et al., 2011). Then, a TF prediction server (http://bioinfo.life.hust.edu.cn/AnimalTFDB/prediction.shtml) was used to identify TFs based on their own protein sequences (Zhang et al., 2015). Moreover, the TF family assignment rules described above were also used for this server, and the TFs identified and obtained from the DEGs were assigned to different TF families for further analysis.

### Validation of Differentially Expressed Genes by Quantitative Real-Time PCR

Quantitative real-time PCR (qRT–PCR) was performed to verify the sequencing results for the 16 selected DEGs. The RNA of each replicate sample was collected using TRIzol Reagent (Invitrogen, Waltham, MA, United States) as described above. First-strand cDNA was synthesized from 1 μg of total RNA with an M-MLV Reverse Transcriptase Kit (Invitrogen, Waltham, MA, United States) following the manufacturer’s instructions. The specific qRT–PCR primers shown in Supplementary Table S1 were designed using Primer 3 software (Untergasser et al., 2012). The reaction mixture consisted of 1 μl (50 ng/μl) of cDNA, 10 μl of iTaq™ Universal SYBR^®^ Green Supermix, 2 μl of each primer, and double-distilled water to a final volume of 20 μl. Reactions were performed in the StepOnePlus Real-Time PCR System (Applied Biosystems) with the following protocol: 95°C for 3 min; 35 cycles of 95°C for 40 s, 60°C for 45 s, and 72°C for 30 s; and 72°C for 10 min for data acquisition. Each sample was tested in triplicate. *β-actin* was selected as the internal reference to normalize the Ct values of each reaction using the 2^−ΔΔCt^ method (Livak and Schmittgen, 2002).

### Interaction Network Map Construction

To further understand the regulatory mechanism of the response to salinity stress, a protein–protein interaction (PPI) network for proteins encoded by DEGs was constructed by mapping the DEGs to the STRING database (https://string-db.org/), and then Cytoscape software was used for the visualization of these potential interactions.

### Excavation of Simple Sequence Repeat Markers

The online identification tool with the Perl script PolyMorphPredict (http://webtom.cabgrid.res.in/polypred/) (Das et al., 2019) was used to identify Simple Sequence Repeat (SSRs) contributing to the salinity tolerance of larvae. The di-, tri-, tetra-, penta-, and hexanucleotide repeat motifs were designed with minimum repeat numbers of 6, 5, 4, 4 and 4 for these SSRs, respectively.

## Results

### Characteristics of the mRNA Library Data

Nine larvae mRNA libraries from the three groups were constructed and sequenced on the Illumina deep-sequencing platform, and a total of approximately 46,331,810 to 56,631,694 raw reads were obtained. After removing ambiguous “N” nucleotides (with a ratio of “N” > 10%) and low-quality sequences (with a quality score <5), there were 47,855,060, 50,862,350 and 50,837,001 clean reads per sample in the S10 group, S13 group and S16 group, respectively, (Table 1). Here, 98% of the clean bases had a quality score of Q20, and approximately 94% of the clean bases had a quality score of Q30, indicating high accuracy of the transcriptome data (Table 2). In addition, a total of 399,066,872 reads were mapped successfully, of which 319,280,127 reads were uniquely mapped. The average mapping ratio is more than 87%.

**TABLE 1:** Statistical data of the transcriptome for the 9 larval libraries under different salinity conditions.

Sample	Raw reads	Clean reads (%)	GC Content (%)	Adapter (%)	Low Quality (%)	Clean Bases (bp)	Q20 (%)	Q30 (%)
S10-1	46,635,272	46,583,226 (99.89%)	50.88	8,286 (0.02%)	85,032 (0.09%)	6,941,153,251	6,816,133,367 (98.2%)	6,567,077,763 (94.61%)
S10-2	4,6,331,810	4,6,251,964 (99.83%)	47.19	12,586 (0.03%)	132,232 (0.14%)	6,914,364,203	6,762,441,233 (97.8%)	6,477,363,358 (93.68%)
S10-3	50,807,028	50,729,990 (99.85%)	50.50	12,052 (0.02%)	127,304 (0.13%)	7,584,024,036	7,427,402,259 (97.93%)	7,128,420,086 (93.99%)
S13-1	44,658,166	44,585,312 (99.84%)	47.88	11,474 (0.03%)	120,496 (0.13%)	6,663,462,165	6,527,603,647 (97.96%)	6,267,495,381 (94.06%)
S13-2	51,542,714	51,465,966 (99.85%)	47.74	13,120 (0.03%)	124,516 (0.12%)	7,697,123,896	7,541,065,942 (97.97%)	7,239,561,433 (94.06%)
S13-3	56,631,694	56,535,772 (99.83%)	47.70	17,040 (0.03%)	155,092 (0.14%)	8,453,065,585	8,264,763,329 (97.77%)	7,908,358,134 (93.56%)
S16-1	50,466,966	50,394,850 (99.86%)	48.98	13,462 (0.03%)	115,004 (0.11%)	7,530,275,740	7,392,018,870 (98.16%)	7,121,365,211 (94.57%)
S16-2	48,559,098	48,475,766 (99.83%)	49.06	15,560 (0.03%)	133,096 (0.14%)	7,250,352,831	7,099,100,643 (97.91%)	6,811,123,919 (93.94%)
S16-3	53,726,210	53,640,388 (99.84%)	48.60	16,136 (0.03%)	136,808 (0.13%)	8,023,805,833	7,865,148,398 (98.02%)	7,559,799,237 (94.22%)

**TABLE 2:** Characteristics of the reads for the 9 samples in this study.

Sample	All Reads number^a^	Unmapped Reads	Unique Mapped Reads	Multiple Mapped Reads	Mapping Ratio (%)	Gene Number
S10-1	39,473,060	5,339,928	29,733,635	4,399,497	86.47	54,960
S10-2	41,572,852	5,531,283	33,392,898	2,648,671	86.69	64,947
S10-3	38,333,450	5,276,011	29,798,176	3,259,263	86.24	58,119
S13-1	40,030,290	5,278,905	31,425,772	3,325,613	86.81	66,521
S13-2	47,364,410	6,637,274	37,683,044	3,044,092	85.99	68,714
S13-3	48,374,566	6,131,917	39,371,388	2,871,261	87.32	66,099
S16-1	47,934,018	5,619,713	39,240,295	3,074,010	88.28	67,825
S16-2	45,446,252	5,239,829	3,7,081,773	3,124,650	88.47	62,422
S16-3	50,537,974	5,885,790	41,553,146	3,099,038	88.35	67,021
Average	44,340,763	5,660,072	35,475,569	3,205,121	87.18	64,069

a the total reads number after ribosomal removal.

### Assembly and Annotation of Unigenes

**FIGURE 1:**
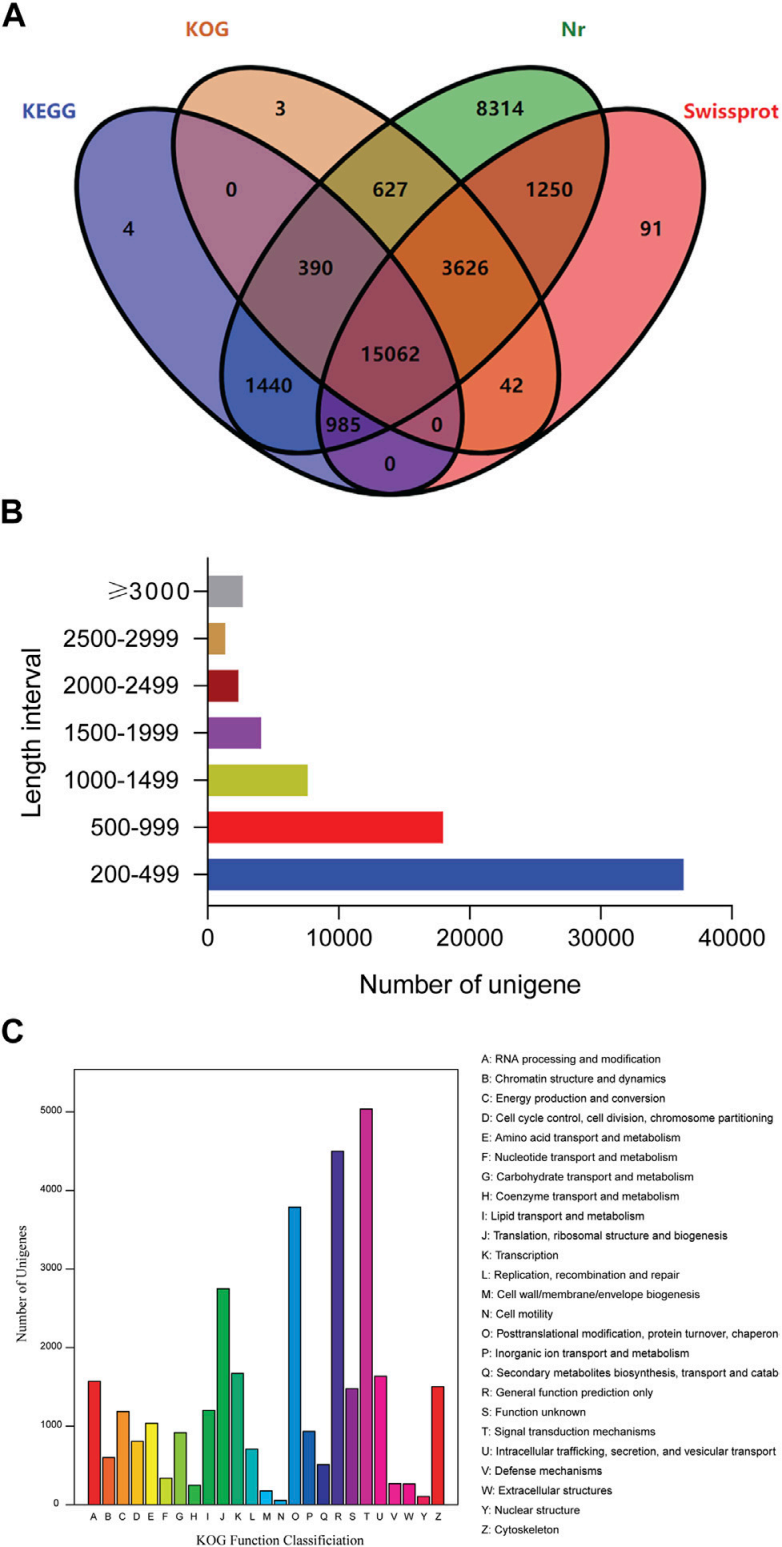
Identification of unigenes. **(A)** Venn diagram showing the common and unique unigenes by four methods, including the KEGG, KOG, Nr and Swiss-protein databases. **(B)** Number of unigenes with different lengths. **(C)** Distribution of unigenes based on functional classification.

The quality of the transcriptome assembly can be evaluated based on the N50 value (sequence length of the shortest transcript at 50% of the total genome length). We identified 72,330 unigenes with an average length of 848 bp, and the N50 value was 1,333 bp. A total of 31,834 unigenes were annotated, accounting for approximately 44% of all the unigenes obtained. Among these, 17,881 could be annotated using the KEGG database (Kyoto Encyclopedia of Genes and Genomes, 24.72%), 19,750 using the KOG database (eukaryotic orthologous groups, 27.31%), 31,694 using the Nr database (nonredundant protein database, 43.82%), and 21,056 using the Swiss-Prot database (Swiss-Protein database, 29.11%). In addition, 4, 3, 8,314, and 91 unigenes were annotated only using the KEGG, KOG, Nr and Swiss-protein databases, respectively (Figure 1A). Among these unigenes, 36,231 (50.22%) were in the 200–499 bp range, 17,956 (24.83%) were in the 500–999 bp range, 11,692 (16.16%) were in the 1–2 kb range, 3,685 (5.09%) were in the 2–3 kb range, and 2,676 (3.70%) were longer than 3 kb (Figure 1B). A total of 19,750 unigenes were divided into 25 KOG classification categories for homologous gene product classification, and one unigene may have multiple functions (Figure 1C). Among the functional classification categories, “signal transduction mechanisms” (15.13%) was the largest group, followed by “general function prediction only” (13.51%) and “posttranslational modification, protein turnover, chaperones” (11.38%), indicating that signal transmission may play an important role in the larval response to salinity changes.

### Identification of Differentially Expressed Genes Under Different Salinity Levels

The gene expression levels for all the samples were evaluated by FPKM values (Supplementary Figure S1A). The correlation of gene expression levels between samples provides an important index for differential expression analysis, and most of the squared Pearson correlation coefficients (*R* ^2^) between the samples were greater than 0.9 (Supplementary Figure S1B), which indicates credible differential expression analysis results.

**FIGURE 2:**
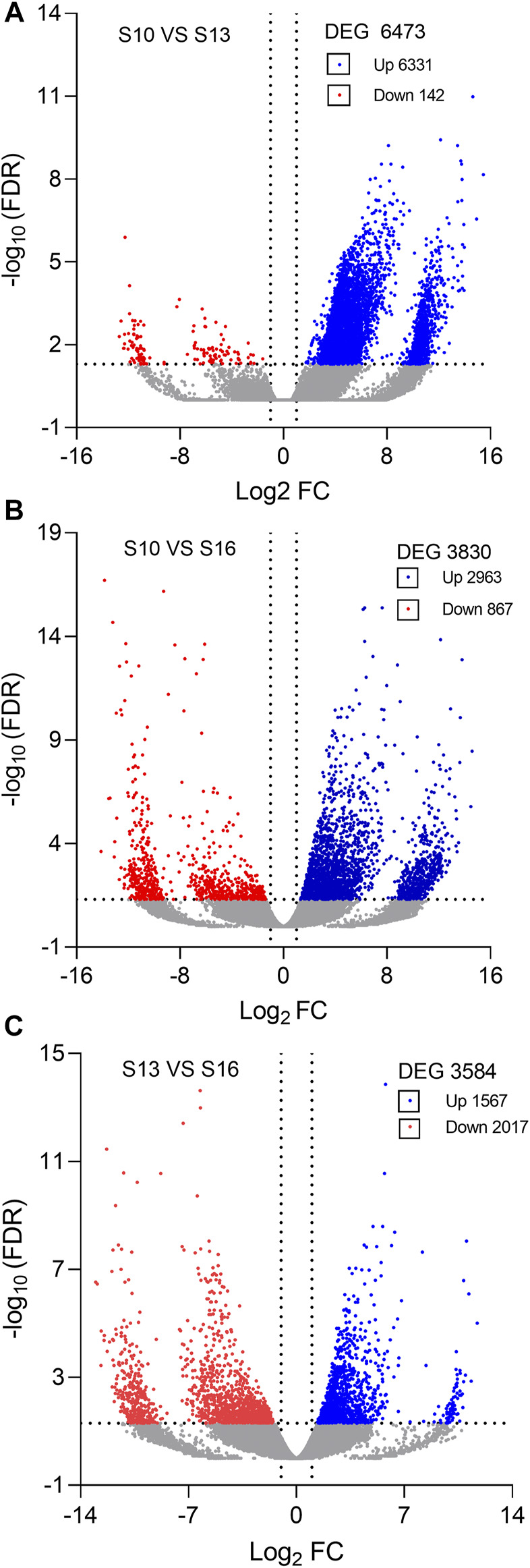
Volcano plots of differentially expressed genes (DEGs) in the S10 vs. S13 comparison **(A)**, S10 vs. S16 comparison **(B)** and S13 vs. S16 comparison **(C)**.

Compared to the S10 condition, a total of 6,473 DEGs were identified under the S13 condition, with 6,331 upregulated genes and 142 downregulated genes (Figure 2A, Supplementary Table S2). In the S10 vs. S16 comparison, 3,830 significant DEGs were identified, including 2,963 upregulated and 867 downregulated genes (Figure 2B, Supplementary Table S3), while in the S13 vs. S16 comparison, 3,584 DEGs were obtained, containing 1,567 upregulated genes and 2017 downregulated genes (Figure 2C, Supplementary Table S4).

### Gene Ontology and Kyoto Encyclopedia of Genes and Genomes Pathway Analysis of Differentially Expressed Genes

**FIGURE 3:**
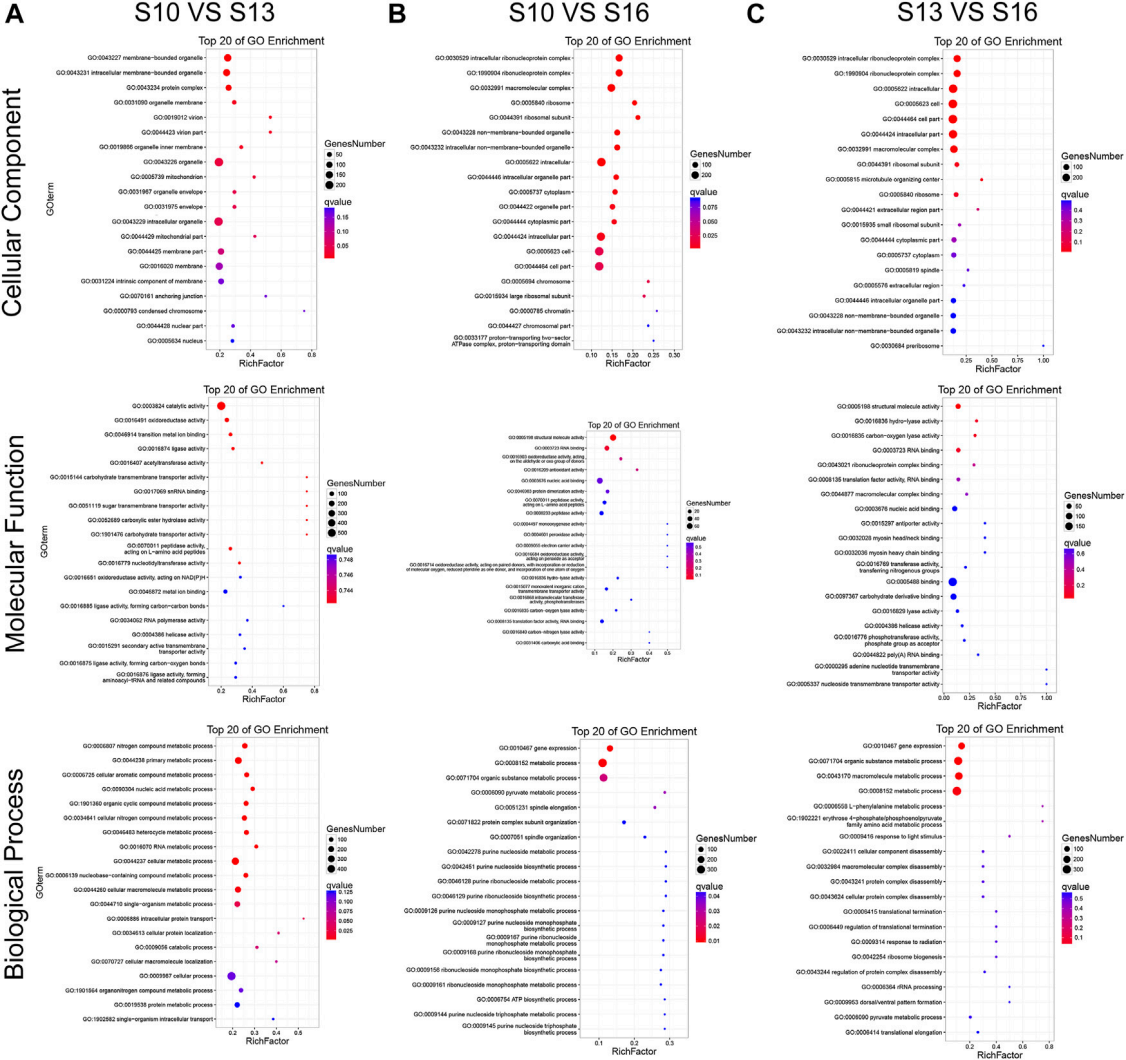
GO enrichment classification of DEGs in the S10 vs. S13 comparison **(A)**, S10 vs. S16 comparison **(B)** and S13 vs. S16 comparison **(C)**.

DEGs can be classified into three major functional categories, “cellular component” (CC), “molecular function” (MF), and “biological process” (BP), based on GO enrichment analysis. In the S10 vs. S13 comparison (Figure 3A, Supplementary Table S5), the most significantly enriched (*p* < 0.05) GO terms were “membrane−bound organelle,” “catalytic activity” and “nitrogen compound metabolic process” in CC, MF, and BP, respectively. The S10 vs. S16 comparison (Figure 3B, Supplementary Table S6) and S13 vs. S16 comparison (Figure 3C, Supplementary Table S7) shared the most significantly enriched (*p* < 0.05) terms, including “intracellular ribonucleoprotein complex,” “structural molecule activity,” and “gene expression”, in CC, MF, and BP, respectively.

**FIGURE 4:**
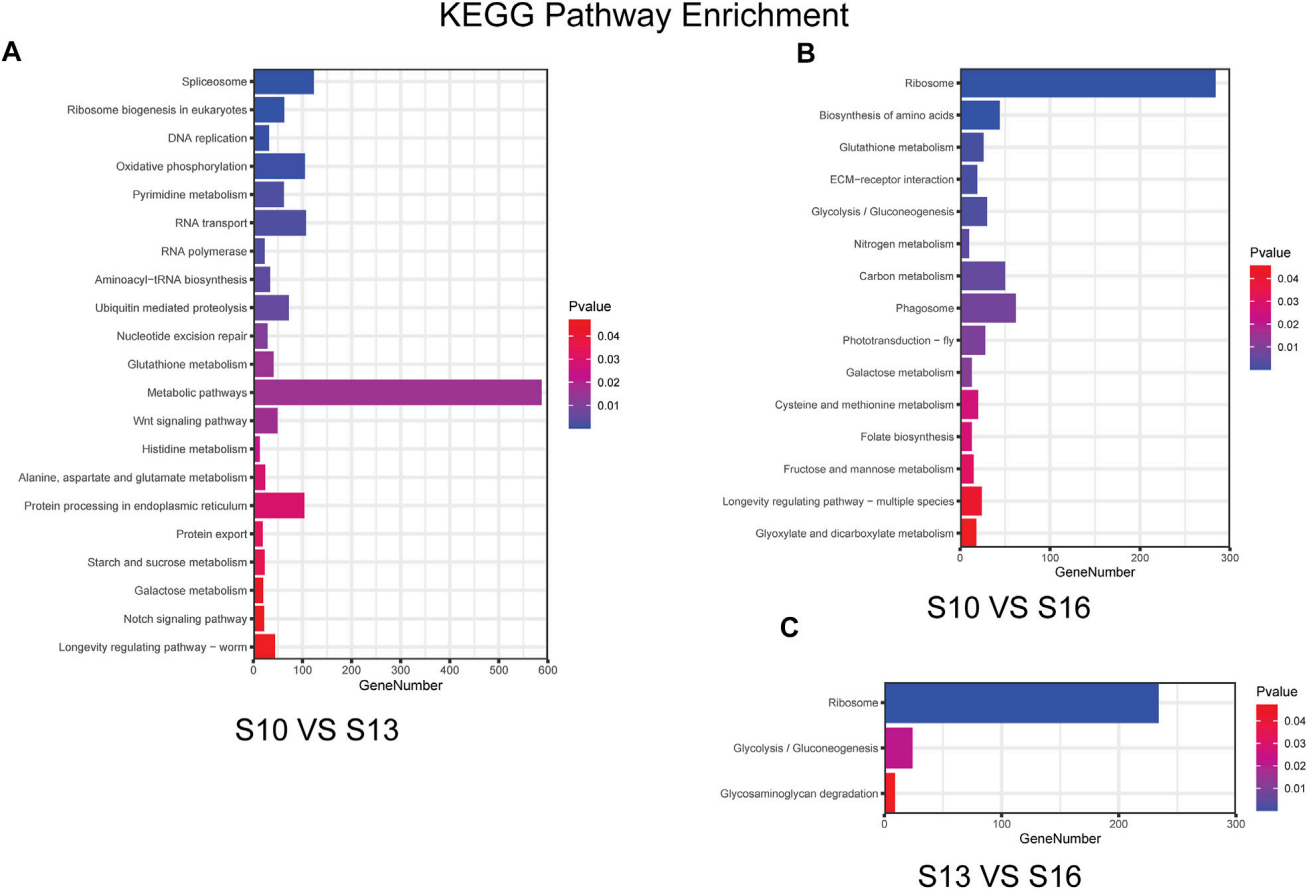
Histogram of the KEGG enrichment results for the S10 vs. S13 comparison **(A)**, S10 vs. S16 comparison **(B)** and S13 vs. S16 comparison **(C)**.

KEGG pathway analysis based on the annotation of the DEGs indicated that 21 KEGG pathways were differentially enriched in the S10 vs. S13 comparison (Figure 4A, Supplementary Table S8), of which the pathway “spliceosome” showed the most differential enrichment and “metabolic pathways” contained the most genes. There were 15 KEGG pathways differentially enriched in the S10 vs. S16 comparison (Figure 4B, Supplementary Table S9), of which the “ribosome” pathway showed the most differential enrichment and had the highest number of genes, which was similar to the results for the S13 vs. S16 comparison (Figure 4C, Supplementary Table S10), where only 3 KEGG pathways were differentially enriched. The S10 vs. S13 comparison and S10 vs. S16 comparison shared the same enrichment for pathways “glutathione metabolism” and “galactose metabolism,” while the pathways “ribosome” and “glycolysis/gluconeogenesis” were found to be enriched in both the S10 vs. S16 comparison and S13 vs. S16 comparison.

### Trend Analysis of the Differentially Expressed Genes

**FIGURE 5:**
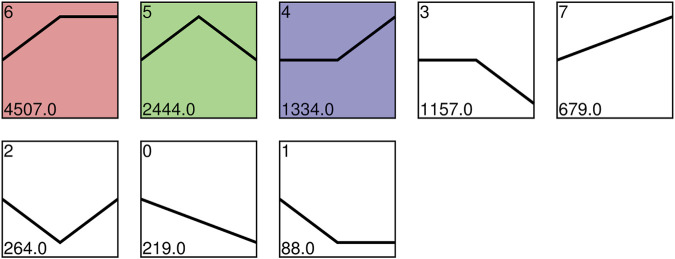
Expression profiles ordered by the number of differentially expressed genes. Colored blocks indicate significant enrichment trends (*p* < 0.05), and different colors indicate different expression trends. The top-left number represents the trend ID. The bottom-left number represents the number of genes.

The expression patterns of the DEGs were explored by trend analyses. A total of 10,692 genes clustered into eight profiles, and profiles 4, 5, and 6 showed significant enrichment (*p* < 0.05), denoted by colored blocks (Figure 5). The expression of 4,507 and 2,444 genes displayed increasing trends before S13 and was stable and downregulated after S13 in profile 6 and profile 5, respectively. In profile 4, the expression of 1,334 genes showed consistent high expression from S10 to S13 but a subsequent increase from S13 to S16.

### Transcription Factor Analysis

A total of 1,535 DEGs were classified into sixty-six kinds of transcription factor families, the top ten of which are shown in Supplementary Figure S2. The transcription factors belonging to the zf-C2H2 family contained the most genes at 847 (55%), followed by the Homeobox family (98 genes, 6.4%), bHLH family (80 genes, 5.2%), and HMG family (55 genes, 3.6%), while the remaining TFs occupied less than 3% of the total number of TFs (Supplementary Table S11).

### Verification of Several Differentially Expressed Genes

**FIGURE 6:**
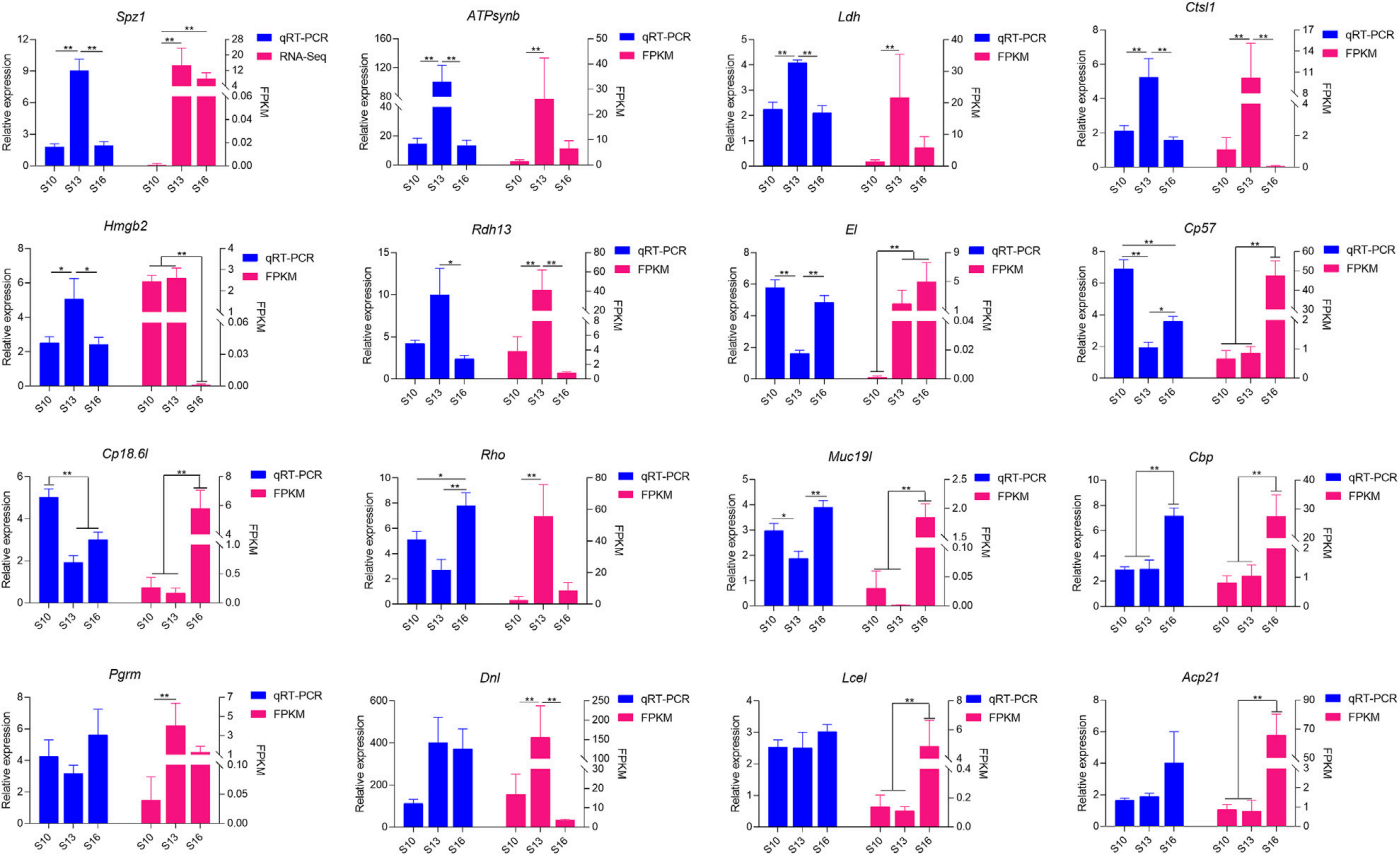
Validation of the 16 DEG profiles by qRT-PCR.

Sixteen differentially expressed genes were randomly selected for validation by transcriptome sequencing (Figure 6). Twelve of these genes were differentially expressed among the three groups; the immune-associated genes SPZ1, LDH, CL1, and HMGB2 and energy metabolism-related genes ATPsynb and RDH13 were all upregulated in the S13 salinity group, while MUC19 and EL1 showed the lowest expression levels. The molting-related genes CP18.6l, CP57 and CBP had different expression patterns among the three groups. The highest expression level was observed in the S10 group for *CP18.6l* and *CP57*, but *CBP* expression was higher in the S16 group. However, there was no difference in the expression of the remaining genes PGRM, DNL, LCEL and ACP21 in the three salinity groups, which was inconsistent with the RNA-seq results.

### Construction of a Protein–Protein Interaction Network Map for the Differentially Expressed Genes

**FIGURE 7:**
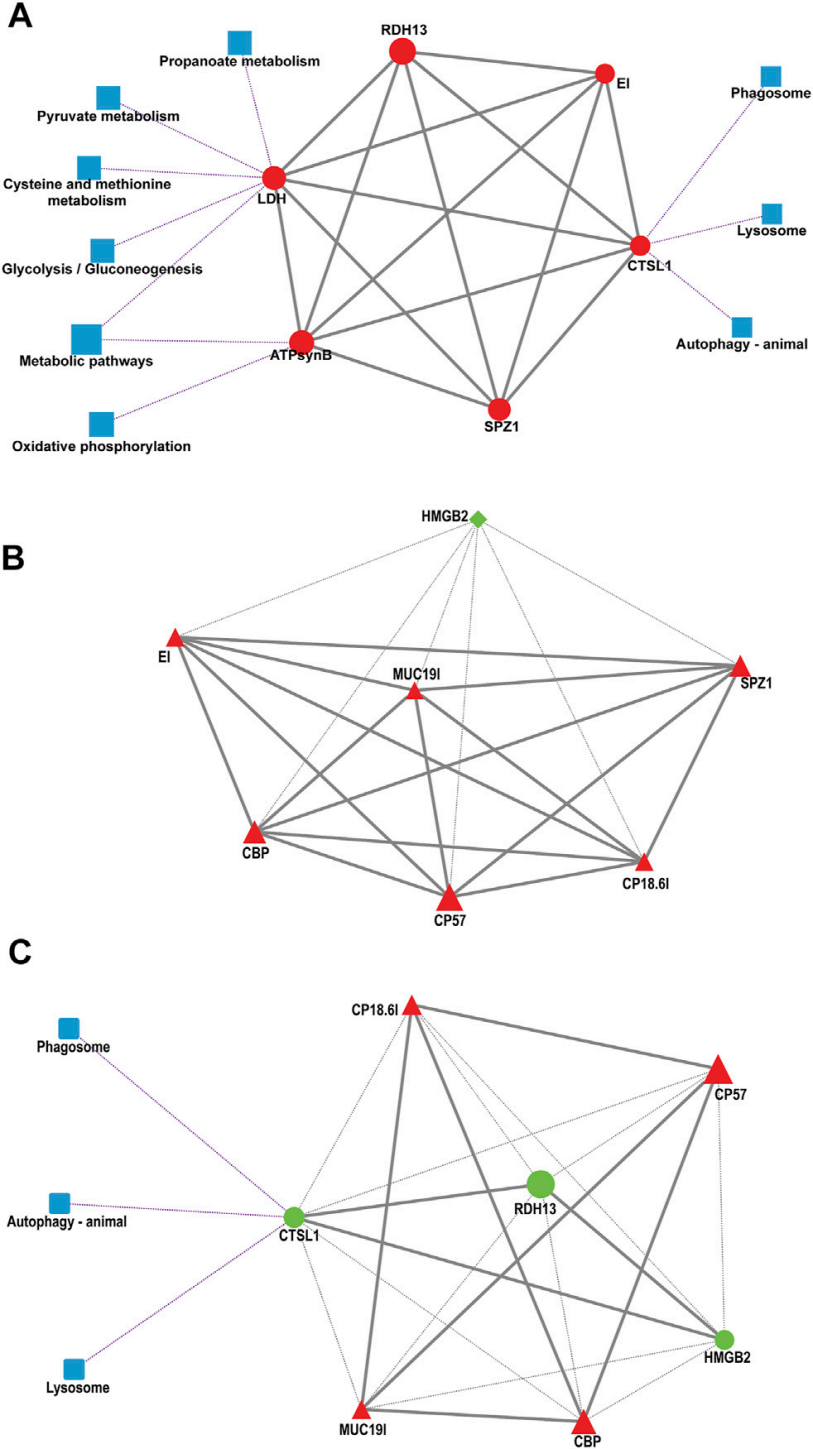
Protein–protein interaction (PPI) networks of DEGs validated by qPCR in the S10 vs. S13 comparison **(A)**, S10 vs. S16 comparison **(B)** and S13 vs. S16 comparison **(C)**. The square represents the pathway that containing genes. The diamond represents the S10 group, the circle represents the S13 group, and the triangle represents the S16 group. The shape of the graph is determined by the unigene RPKM values, where red represents upregulation, green represents downregulation, and blue represents the pathway. The solid line represents a positive correlation, the gray dashed line represents a negative correlation, and the purple dashed line indicates the relationship between the genes and the pathways they are involved in.

To provide further relevant information about the pathways involved in regulating the adaptation of larvae to salinity change, a more comprehensive bioinformatics analysis of protein–protein interaction (PPI) networks was performed for the DEGs validated by qRT–PCR. The PPI network models revealed that there were positive interactions among six genes involved in nine pathways in the S10 vs. S13 comparison (Figure 7A). Here, *LDH* functions in acid metabolism, *ATPsynb* plays a role in oxidative phosphorylation and *CTSL* is involved in immune-related pathways. For the S10 vs. S13 comparison (Figure 7B), *HMGB2* expression was negatively correlated with the other six genes. Interestingly, and in inconsistent with the positive relationship among *CTSL*, *RDH13* and *HMGB2*, the three genes negatively regulated the expression of other genes in the S13 vs. S16 comparison (Figure 7C).

### New EST-SSRs for Larvae Breeding

A total of 7,620 potential EST-SSRs were identified from the 72,330 sequences examined (Table 3, Supplementary Figure S3). Out of the 7,620 SSR motifs identified in *M. rosenbergii*, the most abundant type was trinucleotide (51.1%), followed by di- (40.3%), tetra- (6.3%), hexa- (1.4%), and pentanucleotide motifs (0.8%). AGG/CCT (10.5%) was found to be the most common type of trinucleotide repeat, followed by AAG/CTT (10.45%) and AGC/CTG (9.4%). Among the dinucleotide repeats, the most frequent motif was AG/CT (23.9%), followed by AC/GT (2.39%) and AT/AT (2.7%).

**TABLE 3:** Parameters of the SSRs obtained from the sequences examined in the transcriptome.

Stat item	Number
Total number of sequences examined	72,330
Total size of examined sequences (bp)	61,349,492
Total number of identified SSRs	7,620
Number of SSR containing sequences	5,703
Number of sequences containing more than 1 SSR	1,263
Number of SSRs present in compound formation	856
Di-nucleotide	3,071
Tri-nucleotide	3,895
Tetra-nucleotide	480
Penta-nucleotide	61
Hexa-nucleotide	113

## Discussion

Salinity is one of the most crucial environmental factors affecting the lives of aquatic animals, especially decapod crustaceans that cannot live in fresh water during early ontogenesis (Anger, 2003). Salinity changes can cause a series of physiological reactions, including osmotic regulation, ion transportation and respiratory metabolism, which maintain bodily homeostasis (Laiz-Carrión et al., 2005; Whittamore, 2012; Giffard-Mena et al., 2020; Tian et al., 2020). To understand the mechanism underlying larval adaptation to salinity variation under artificial breeding conditions, total RNA extraction, library construction and sequencing were performed on larvae cultured under S10, S13, and S16 conditions. We eventually obtained some differentially enriched pathways among the groups *via* GO and KEGG pathway analysis. Consistent with work in *Litopenaeus vannamei* (Hu et al., 2015), the MAPK signaling pathway and energy-related pathways, such as glycolysis/gluconeogenesis and oxidative phosphorylation, were also differentially enriched in our research under salinity challenge. Detailed information on these DEGs was obtained through gene annotation, and most are involved in some key biological functions. PPI networks showed that some DEGs influence the response to salinity changes in *M. rosenbergii* by directly or indirectly regulating the expression of other genes. To provide more information about the mechanism underlying the response to salinity change, several key functional categories are discussed below.

### Osmoregulation and Energy Metabolism

The osmotic regulation ability of aquatic animals is a key factor for their adaptation to waters with varying salinity (Malik and Kim, 2021). Several studies have demonstrated that a salinity of approximately 14‰ is the optimal condition for several aquatic invertebrate animals (Cheng et al., 2003; Huang et al., 2019b), which was similar to our previous research showing a higher metamorphosis rate detected under a salinity of 13‰ than under a salinity of 10‰ or a salinity of 16‰ (Wei et al., 2021). The energy used for osmotic pressure adjustment requires 20–50% of the total energy of the organism (Ern et al., 2014), which is mainly applied for ion transportation and acid-base balance (Sébert et al., 1997). Genes associated with ATP synthase were upregulated, such as F0-ATP synthase b-chain *ATPsynb* in the S13 group. ATP synthase is a crucial enzyme in the oxidative phosphorylation pathway that transfers energy from the substrate to ATP produced in the inner mitochondrial membrane (Silva-Marrero et al., 2017). Another study indicated that high salinity can result in increased ATPase activity in shrimp (Hurtado et al., 2007). These findings suggested that salinity can influence osmoregulation and energy metabolism. In addition, *ATPsynb* was also reported to function as an antiviral by interacting with VP292, an envelope protein of WSVV, implying an increase in the demand for energy during the process of fighting viral infection (Bourchookarn et al., 2008; Li et al., 2013). In this study, we found that *ATPsynb* is also involved in oxidative phosphorylation *via* PPI network analysis. Therefore, *ATPsynb* was deduced to be the critical molecule regulating osmoregulation and energy metabolism for adaptation to salinity change in *M. rosenbergii* in our research.

### Metamorphosis and Molting

Metamorphosis is a key process in the growth, development, and reproduction of crustaceans that occurs early in the lifetime of the organism and is affected by several environmental factors (e.g., temperature, light, and salinity) (Gardner and Maguire, 1998; Chen and Chen, 2002; Hoang et al., 2003; Gong et al., 2015; Andersen et al., 2022). Molting is one of the most significant characteristics of metamorphosis in arthropods (Minelli et al., 2006). Limited by the exoskeleton, during growth and development, arthropods must undergo molting, which is characterized by the shedding of old epidermis and regeneration of new epidermis that requires the coregulation of endogenous hormones and exogenous factors (Hopkins and Kramer, 1992; Mun et al., 2015).

A previous report showed that in arthropods such as insects, cuticular protein is cross-linked by quinones or quinone methides during cuticle sclerotization, which are required for molting (Mun et al., 2015). Chitin-binding protein serves as the main component of the epidermis and is essential to maintaining and regulating the functions of these extracellular structures. It was reported that the chitin binding process was enriched during molting of *Portunus trituberculatus* (Lv et al., 2017); this feature is comparable to the carapace of other crustaceans. In our research, several genes associated with molting, *CP18.6l*, *CP57* and *CBP*, were screened and validated, and they were differentially expressed among the three groups. The transcript levels of *CP18.6L* and *CP57* decreased with increased salinity, while the expression trend of *CBP* was the opposite. Previous studies suggested that salinity can influence the survival of megalopa through metamorphosis to the first juvenile crab stage in the freshwater-tolerant sesarmid crab *Armases roberti* (Torres et al., 2006), and a proper salinity level benefited embryonic development and larval survival at metamorphosis (Madrones-Ladja, 2002). Together with these findings, we speculated that the three kinds of chitin-binding proteins may be involved in molting in a salinity-dependent manner during early development and metamorphosis of larvae.

Retinol dehydrogenase can catalyze vitamin A into retinoic acid, which is essential to embryonic development (Tanaka et al., 2005; Duester, 2008), and the retinoic acid signaling pathway containing retinol dehydrogenase 14 is reported to be one of the key regulatory factors in modulating eye migration during Japanese flounder metamorphosis (Shao et al., 2017). Retinol dehydrogenase 13 (*Rdh13*) has been previously identified as a short-chain dehydrogenase/reductase located on the outer side of the inner mitochondrial membrane, indicating that it may be involved in protecting mitochondria against oxidative stress (Belyaeva et al., 2008). In the current study, *Rdh13* was expressed at the highest level in the S13 group and positively regulated the expression of *ATPsynb*, which is involved in oxidative phosphorylation, in the PPI network model. Combined with our recent research results, a higher metamorphosis rate was detected under 13‰ salinity, indicating that *Rdh13* may play an important role in maintaining the metamorphosis of crustaceans by protecting mitochondria against oxidative stress by adjusting the *ATPsynb* transcript level.

### Immune Response

It is widely recognized that aquatic animals usually develop an immune response to adapt to changeable environments (e.g., salinity changes) under the stimulation of unfavorable environmental factors. Similar responses have been reported in *Cardisoma armatum* (Wu et al., 2021), which revealed that salinity variation can increase the expression of immune-related genes. In the present study, a large number of DEGs related to immune-related pathways, such as spermatogenic leucine zipper 1 (Spz1), lactate dehydrogenase (*Ldh*), extensin-like (*El*), high mobility group protein (*Hmg*), mucin gene 19-like (*Muc19l*), and cathepsin L (*Ctsl*), were differentially enriched among these groups.

The BHLH-zip protein is implicated in cell growth and differentiation and plays a vital role in tumorigenesis. The Spz1 gene encodes a bHLH-zip transcription factor that functions in the mitogen-activated protein kinase (MAPK) signaling pathway. It has been revealed that a reduction in Spz1 levels based on RNA interference decreased embryonic carcinoma cell proliferation (Hsu et al., 2005). *Ldh* is an oxidoreductase that is involved in the process of carbon hydrate glycolysis and has been reported to function in hypertension and oxidative stress (Mokwatsi et al., 2016). It can help the innate immune system fight off *Staphylococcus aureus* after nitric oxide induction (Richardson et al., 2008), whereas increased expression of lactate dehydrogenase after functional overload in rat soleus muscle implied that the stress response may result in a high level of lactate dehydrogenase (Washington et al., 2004). In a recent report, elevated lactate dehydrogenase caused a significant reduction in lifespan by causing Drosophila brain neurodegeneration (Long et al., 2020).

Moreover, extensin-like proteins can enhance drought resistance of wheat (KeskİN, 2019). Hmgb1/2 inhibits genotoxic stress in polyglutamine diseases by interacting with mutant ataxin-1 and huntingtin protein (Qi et al., 2007); suppressing Hmgb2 expression causes gastric cancer cells to be less sensitive to chemotherapy (An et al., 2015). Mucins and mucin-like molecules provide physical protection for organisms from external pathogens (Martín et al., 2019), and MUC19 protects the ocular surface from shear force damage, desiccation, and microbial invasion (Yu et al., 2008). *Ctsl* is one of the crucial enzyme superfamilies and is involved in the resistance to pathogen infection (Dai et al., 2017). Increased numbers of eosinophils were found after vaccination, indicating that recombinant cysteine proteinase (cathepsin L1) can promote immunity in rats and protect cattle from natural infection with *Fasciola hepatica* (Kęsik et al., 2007; Golden et al., 2010). *Ctsl* was also detected to be involved in immune-related pathways by PPI network model analysis in this study. Overall, these immune-related genes displayed different up- and downregulated transcript levels, showing that they played different regulatory roles in responding to salinity variation by increasing the immune-related pathway, which needs further study.

## Conclusion

In this study, transcriptome analysis was performed to investigate the molecular response to exposure to different salinity levels under the S10, S13 and S16 treatments. Differentially expressed genes and related pathways among the three salinity levels that were involved in osmoregulation and energy metabolism, metamorphosis and molting, and immune response were identified. PPI network analysis indicated the important roles of interactions among the DEGs in regulating the adaptation of larvae to salinity challenge. It is crucial to understand this mechanism to provide new methods to improve the survival rate and metamorphosis rate and increase the production of *M. rosenbergii*.

## Data Availability

The datasets presented in this study can be found in online repositories with the names of the repository/repositories and accession number(s) are below: NCBI (accession: PRJNA822661).

## Supplementary Material

Supplementary Figure S1 Expression levels of 9 larval samples. **(A)** Boxplots of the 9 transcripts based on fragments per kilobase of transcript per million mapped fragments (FPKM). **(B)** Heatmap plot showing the correlation of gene expression levels among the samples.

Supplementary Figure S2 Transcription factor family classification for the 1535 DEGs from the complete transcriptome.

Supplementary Figure S3 The proportion of different motifs across all SSRs.

## References

[B1] Alam M. R. Sharmin S. Islam S. M. Alam M. A. Ehiguese F. O. Pattadar S. N. (2020). Salinity Intrusion Affects Early Development of Freshwater Aquaculture Species Pabda, Ompok Pabda. Aquac. Rep. 18, 100476. 10.1016/j.aqrep.2020.100476

[B2] An Y. Zhang Z. Shang Y. Jiang X. Dong J. Yu P. (2015). miR-23b-3p Regulates the Chemoresistance of Gastric Cancer Cells by Targeting ATG12 and HMGB2. Cell Death Dis 6 (5), e1766. 10.1038/cddis.2015.123 25996293 PMC4669702

[B3] Andersen Ø. Johnsen H. Wittmann A. C. Harms L. Thesslund T. Berg R. S. (2022). De Novo transcriptome Assemblies of Red king Crab (*Paralithodes Camtschaticus*) and Snow Crab (*Chionoecetes Opilio*) Molting Gland and Eyestalk Ganglia - Temperature Effects on Expression of Molting and Growth Regulatory Genes in Adult Red king Crab. Comp. Biochem. Physiol. B: Biochem. Mol. Biol. 257, 110678. 10.1016/j.cbpb.2021.110678 34655763

[B4] Anger K. (2003). Salinity as a Key Parameter in the Larval Biology of Decapod Crustaceans. Invertebrate Reprod. Dev. 43 (1), 29–45. 10.1080/07924259.2003.9652520

[B5] Belyaeva O. V. Korkina O. V. Stetsenko A. V. Kedishvili N. Y. (2008). Human Retinol Dehydrogenase 13 (RDH13) Is a Mitochondrial Short-Chain Dehydrogenase/reductase with a Retinaldehyde Reductase Activity. FEBS J. 275 (1), 138–147. 10.1111/j.1742-4658.2007.06184.x 18039331 PMC2573044

[B6] Bourchookarn A. Havanapan P.-O. Thongboonkerd V. Krittanai C. (2008). Proteomic Analysis of Altered Proteins in Lymphoid Organ of Yellow Head Virus Infected *Penaeus monodon* . Biochim. Biophys. Acta (BBA) - Proteins Proteomics 1784 (3), 504–511. 10.1016/j.bbapap.2007.12.006 18206130

[B7] Cabau C. Escudié F. Djari A. Guiguen Y. Bobe J. Klopp C. (2016). Compacting and Correcting Trinity and Oases RNA-Seq De Novo Assemblies. PeerJ 5, e2988. 10.7717/peerj.2988 PMC5316280 28224052

[B8] Chand B. K. Trivedi R. K. Dubey S. K. Rout S. K. Beg M. M. Das U. K. (2015). Effect of salinity on survival and growth of giant freshwater prawn *Macrobrachium rosenbergii* (de Man). Aquac. Rep. 2, 26–33. 10.1016/j.aqrep.2015.05.002

[B9] Chen S. Zhou Y. Chen Y. Gu J. (2018). Fastp: an Ultra-fast All-In-One FASTQ Preprocessor. Bioinformatics 34, i884–i890. 10.1093/bioinformatics/bty560 30423086 PMC6129281

[B10] Chen Y.-H. Chen I.-M. (2002). Effects of Temperature and Salinity on the Metamorphosis of Nauplius of a Planktonic Shrimp *Acetes Intermedius* Omori, 1975. Fish. Sci. 68 (1), 117–122. 10.1046/j.1444-2906.2002.00396.x

[B11] Cheng W. Liu C.-H. Cheng C.-H. Chen J.-C. (2003). Osmolality and Ion Balance in Giant River Prawn *Macrobrachium Rosenbergii* Subjected to Changes in Salinity: Role of Sex. Aqua. Res. 34 (7), 555–560. 10.1046/j.1365-2109.2003.00853.x

[B12] Choi C. Y. An K. W. An M. I. (2008). Molecular Characterization and mRNA Expression of Glutathione Peroxidase and Glutathione S-Transferase during Osmotic Stress in Olive Flounder (*Paralichthys olivaceus*). Comp. Biochem. Physiol. A: Mol. Integr. Physiol. 149 (3), 330–337. 10.1016/j.cbpa.2008.01.013 18302988

[B13] Cui Q. Qiu L. Yang X. Shang S. Yang B. Chen M. (2019). Transcriptome Profiling of the Low-Salinity Stress Responses in the Gills of the Juvenile *Pseudopleuronectes yokohamae* . Comp. Biochem. Physiol. D: Genomics Proteomics 32, 100612. 10.1016/j.cbd.2019.100612 31387066

[B14] Dai L.-S. Chu S.-H. Yu X.-M. Li Y.-Y. (2017). A Role of Cathepsin L Gene in Innate Immune Response of Crayfish ( *Procambarus clarkii* ). Fish Shellfish Immunol. 71, 246–254. 10.1016/j.fsi.2017.10.021 29032038

[B15] Das R. Arora V. Jaiswal S. Iquebal M. Angadi U. Fatma S. (2019). PolyMorphPredict: a Universal Web-Tool for Rapid Polymorphic Microsatellite Marker Discovery from Whole Genome and Transcriptome Data. Front. Plant Sci. 9, 1–10. 10.3389/fpls.2018.01966 PMC6337687 30687361

[B16] Dawood M. A. O. Noreldin A. E. Sewilam H. (2021). Long Term Salinity Disrupts the Hepatic Function, Intestinal Health, and Gills Antioxidative Status in Nile tilapia Stressed with Hypoxia. Ecotoxicology Environ. Saf. 220, 112412. 10.1016/j.ecoenv.2021.112412 34119925

[B17] Duester G. (2008). Retinoic Acid Synthesis and Signaling during Early Organogenesis. Cell 134 (6), 921–931. 10.1016/j.cell.2008.09.002 18805086 PMC2632951

[B18] Ern R. Huong D. T. T. Cong N. V. Bayley M. Wang T. (2014). Effect of Salinity on Oxygen Consumption in Fishes: a Review. J. Fish. Biol. 84 (4), 1210–1220. 10.1111/jfb.12330 24665828

[B19] Ernst J. Bar-Joseph Z. (2006). STEM: a Tool for the Analysis of Short Time Series Gene Expression Data. BMC bioinformatics 7, 191. 10.1111/jfb.1233010.1186/1471-2105-7-191 16597342 PMC1456994

[B20] FAO (2021). Food and. Rome, Italy: Agriculture Organization of the United Nations.

[B21] Finn R. D. Clements J. Eddy S. R. (2011). HMMER Web Server: Interactive Sequence Similarity Searching. Nucleic Acids Res. 39, W29–W37. 10.1093/nar/gkr367 21593126 PMC3125773

[B22] Gardner C. Maguire G. B. (1998). Effect of Photoperiod and Light Intensity on Survival, Development and Cannibalism of Larvae of the Australian Giant Crab *Pseudocarcinus Gigas* (Lamarck). Aquaculture 165 (1), 51–63. 10.1016/s0044-8486(98)00245-2

[B23] Giffard-Mena I. Hernández-Montiel Á. H. Pérez-Robles J. David-True C. (2020). Effects of Salinity on Survival and Plasma Osmolarity of *Totoaba Macdonaldi* Eggs, Larvae, and Juveniles. J. Exp. Mar. Biol. Ecol. 526, 151339. 10.1016/j.jembe.2020.151339

[B24] Golden O. Flynn R. J. Read C. Sekiya M. Donnelly S. M. Stack C. (2010). Protection of Cattle against a Natural Infection of Fasciola Hepatica by Vaccination with Recombinant Cathepsin L1 (rFhCL1). Vaccine 28 (34), 5551–5557. 10.1016/j.vaccine.2010.06.039 20600503

[B25] Gong J. Yu K. Shu L. Ye H. Li S. Zeng C. (2015). Evaluating the Effects of Temperature, Salinity, Starvation and Autotomy on Molting success, Molting Interval and Expression of Ecdysone Receptor in Early Juvenile Mud Crabs, *Scylla Paramamosain* . J. Exp. Mar. Biol. Ecol. 464, 11–17. 10.1016/j.jembe.2014.12.008

[B26] Hoang T. Barchiesis M. Lee S. Y. Keenan C. P. Marsden G. E. (2003). Influences of Light Intensity and Photoperiod on Moulting and Growth of *Penaeus Merguiensis* Cultured under Laboratory Conditions. Aquaculture 216 (1), 343–354. 10.1016/s0044-8486(02)00460-x

[B27] Hopkins T. L. Kramer K. J. (1992). Insect Cuticle Sclerotization. Annu. Rev. Entomol. 37, 273–302. 10.1146/annurev.en.37.010192.001421

[B28] Hsu S.-H. Hsieh-Li H.-M. Huang H.-Y. Huang P.-H. Li H. (2005). bHLH-Zip Transcription Factor Spz1 Mediates Mitogen-Activated Protein Kinase Cell Proliferation, Transformation, and Tumorigenesis. Cancer Res. 65 (10), 4041–4050. 10.1158/0008-5472.can-04-3658 15899793

[B29] Hu D. Pan L. Zhao Q. Ren Q. (2015). Transcriptomic Response to Low Salinity Stress in Gills of the Pacific white Shrimp, *Litopenaeus Vannamei* . Mar. Genomics 24, 297–304. 10.1016/j.margen.2015.07.003 26210687

[B30] Huang M. Dong Y. Zhang Y. Chen Q. Xie J. Xu C. (2019a). Growth and Lipidomic Responses of Juvenile Pacific White Shrimp *Litopenaeus Vannamei* to Low Salinity. Front. Physiol. 10, 1087. 10.3389/fphys.2019.01087 31507450 PMC6716509

[B31] Huang Y. H. Zhang M. Li Y. M. Wu D. L. Liu Z. Q. Jiang Q. C. (2019b). Effects of Salinity Acclimation on the Growth Performance, Osmoregulation and Energy Metabolism of the oriental River Prawn, *Macrobrachium Nipponense* (De Haan). Aquac. Res. 50 (2), 685–693. 10.1111/are.13950

[B32] Hurtado M. A. Racotta I. S. Civera R. Ibarra L. Hernández-Rodríguez M. Palacios E. (2007). Effect of Hypo- and Hypersaline Conditions on Osmolality and Na^+^/K^+^-ATPase Activity in Juvenile Shrimp (*Litopenaeus Vannamei*) Fed Low- and High-HUFA Diets. Comp. Biochem. Physiol. A. Mol. Integr. Physiol. 147 (3), 703–710. 10.1016/j.cbpa.2006.07.002 16935535

[B33] Kęsik M. Jedlina-Panasiuk L. Kozak-Cięszczyk M. Płucienniczak A. Wędrychowicz H. (2007). Enteral Vaccination of Rats against Fasciola Hepatica Using Recombinant Cysteine Proteinase (Cathepsin L1). Vaccine 25 (18), 3619–3628. 10.1016/j.vaccine.2007.01.057 17289224

[B34] Keski̇n B. C. (2019). Quantitative mRNA Expression Profiles of Germin-like and Extensin-like Proteins under Drought Stress in *Triticum aestivum* . Int. J. Life Sci. Biotechnol. 2, 95–107. 10.38001/ijlsb.566942

[B35] Laiz-Carrión R. Sangiao-Alvarellos S. Guzmán J. M. Martín del Río M. P. Soengas J. L. Mancera J. M. (2005). Growth Performance of Gilthead Sea Bream *Sparus Aurata* in Different Osmotic Conditions: Implications for Osmoregulation and Energy Metabolism. Aquaculture 250 (3), 849–861. 10.1016/j.aquaculture.2005.05.021

[B36] Li Q. Liu Q.-h. Huang J. (2013). F0ATP Synthase B-Chain of Litopenaeus Vannamei Involved in White Spot Syndrome Virus Infection. Virus Genes 47 (1), 42–48. 10.1007/s11262-013-0907-1 23558437

[B37] Liu Z. Ma A. Yuan C. Zhao T. Chang H. Zhang J. (2021). Transcriptome Analysis of Liver Lipid Metabolism Disorders of the Turbot *Scophthalmus maximus* in Response to Low Salinity Stress. Aquaculture 534, 736273. 10.1016/j.aquaculture.2020.736273

[B38] Livak K. J. Schmittgen T. D. (2001). Analysis of Relative Gene Expression Data Using Real-Time Quantitative PCR and the 2−ΔΔCT Method. Methods 25, 402–408. 10.1006/meth.2001.1262 11846609

[B39] Long D. M. Frame A. K. Reardon P. N. Cumming R. C. Hendrix D. A. Kretzschmar D. (2020). Lactate Dehydrogenase Expression Modulates Longevity and Neurodegeneration in *Drosophila melanogaster* . Aging 12 (11), 10041–10058. 10.18632/aging.103373 32484787 PMC7346061

[B40] Love M. I. Huber W. Anders S. (2014). Moderated Estimation of Fold Change and Dispersion for RNA-Seq Data with DESeq2. Genome Biol. 15 (12), 550. 10.1186/s13059-014-0550-8 25516281 PMC4302049

[B41] Lv J. Zhang L. Liu P. Li J. (2017). Transcriptomic Variation of Eyestalk Reveals the Genes and Biological Processes Associated with Molting in *Portunus Trituberculatus* . PLoS One 12 (4), e0175315. 10.1371/journal.pone.0175315 28394948 PMC5386282

[B42] Madrones-Ladja J. A. (2002). Salinity Effect on the Embryonic Development, Larval Growth and Survival at Metamorphosis of *Placuna Placenta* Linnaeus (1758). Aquaculture 214 (1-4), 411–418. 10.1016/s0044-8486(02)00401-5

[B43] Malik A. Kim C.-B. (2021). Role of Transportome in the Gills of Chinese Mitten Crabs in Response to Salinity Change: a Meta-Analysis of RNA-Seq Datasets. Biology 10 (1), 39. 10.3390/biology10010039 33430106 PMC7827906

[B44] Minelli A. Brena C. Deflorian G. Maruzzo D. Fusco G. (2006). From Embryo to Adult-Beyond the Conventional Periodization of Arthropod Development. Dev. Genes Evol. 216 (7), 373–383. 10.1007/s00427-006-0075-6 16670874

[B45] Mokwatsi G. G. Schutte A. E. Kruger R. (2016). A Biomarker of Tissue Damage, Lactate Dehydrogenase, Is Associated with Fibulin-1 and Oxidative Stress in Blacks: the SAfrEIC Study. Biomarkers 21 (1), 48–55. 10.3109/1354750x.2015.1118532 26631026

[B46] Mun S. Young Noh M. Dittmer N. T. Muthukrishnan S. Kramer K. J. Kanost M. R. (2015). Cuticular Protein with a Low Complexity Sequence Becomes Cross-Linked during Insect Cuticle Sclerotization and Is Required for the Adult Molt. Sci. Rep. 5 (1), 10484. 10.1038/srep10484 25994234 PMC4440208

[B47] New M. B. (2005). Freshwater Prawn Farming: Global Status, Recent Research and a Glance at the Future. Aquac. Res. 36 (3), 210–230. 10.1111/j.1365-2109.2005.01237.x

[B48] Ni Q. Li W. Liang X. Liu J. Ge H. Dong Z. (2021). Gill Transcriptome Analysis Reveals the Molecular Response to the Acute Low-Salinity Stress in *Cyclina Sinensis* . Aquac. Rep. 19, 100564. 10.1016/j.aqrep.2020.100564

[B49] Ogata H. Goto S. Sato K. Fujibuchi W. Bono H. Kanehisa M. (1999). KEGG: Kyoto Encyclopedia of Genes and Genomes. Nucleic Acids Res. 27, 29–34. 10.1093/nar/27.1.29 9847135 PMC148090

[B50] Perry S. F. Rivero-Lopez L. McNeill B. Wilson J. (2006). Fooling a Freshwater Fish: How Dietary Salt Transforms the Rainbow trout Gill into a Seawater Gill Phenotype. J. Exp. Biol. 209 (23), 4591–4596. 10.1242/jeb.02558 17114394

[B51] Pertea M. Kim D. Pertea G. M. Leek J. T. Salzberg S. L. (2016). Transcript-level Expression Analysis of RNA-Seq Experiments with HISAT, StringTie and Ballgown. Nat. Protoc. 11 (9), 1650–1667. 10.1038/nprot.2016.095 27560171 PMC5032908

[B52] Pinzón Martín S. Seeberger P. H. Varón Silva D. (2019). Corrigendum: Mucins and Pathogenic Mucin-like Molecules Are Immunomodulators during Infection and Targets for Diagnostics and Vaccines. Front. Chem. 7, 710. 10.3389/fchem.2019.00846 31696111 PMC6817596

[B53] Qi M.-L. Tagawa K. Enokido Y. Yoshimura N. Wada Y.-i. Watase K. (2007). Proteome Analysis of Soluble Nuclear Proteins Reveals that HMGB1/2 Suppress Genotoxic Stress in Polyglutamine Diseases. Nat. Cel Biol. 9 (4), 402–414. 10.1038/ncb1553 17384639

[B54] Richardson A. R. Libby S. J. Fang F. C. (2008). A Nitric Oxide-Inducible Lactate Dehydrogenase Enables *Staphylococcus aureus* to Resist Innate Immunity. Science 319 (5870), 1672–1676. 10.1126/science.1155207 18356528

[B55] Sébert P. Simon B. Péqueux A. (1997). Effects of Hydrostatic Pressure on Energy Metabolism and Osmoregulation in Crab and Fish. Comp. Biochem. Physiol. A. Mol. Integr. Physiol. 116 (4), 281–290. 10.1016/s0300-9629(96)00353-2

[B56] Shao C. Bao B. Xie Z. Chen X. Li B. Jia X. (2017). The Genome and Transcriptome of Japanese Flounder Provide Insights into Flatfish Asymmetry. Nat. Genet. 49 (1), 119–124. 10.1038/ng.3732 27918537

[B57] Silva-Marrero J. I. Sáez A. Caballero-Solares A. Viegas I. Almajano M. P. Fernández F. (2017). A Transcriptomic Approach to Study the Effect of Long-Term Starvation and Diet Composition on the Expression of Mitochondrial Oxidative Phosphorylation Genes in Gilthead Sea Bream (*Sparus Aurata*). BMC Genomics 18, 768. 10.1186/s12864-017-4148-x 29020939 PMC5637328

[B58] Tanaka Y. Okada Y. Hirokawa N. (2005). FGF-induced Vesicular Release of Sonic Hedgehog and Retinoic Acid in Leftward Nodal Flow Is Critical for Left-Right Determination. Nature 435 (7039), 172–177. 10.1038/nature03494 15889083

[B59] Tian L. Tan P. Yang L. Zhu W. Xu D. (2020). Effects of Salinity on the Growth, Plasma Ion Concentrations, Osmoregulation, Non-specific Immunity, and Intestinal Microbiota of the Yellow Drum (*Nibea Albiflora*). Aquaculture 528, 735470. 10.1016/j.aquaculture.2020.735470

[B60] Torres G. Anger K. Giménez L. (2006). Effects of Reduced Salinities on Metamorphosis of a Freshwater-Tolerant Sesarmid Crab, *Armases Roberti*: Is Upstream Migration in the Megalopa Stage Constrained by Increasing Osmotic Stress? J. Exp. Mar. Biol. Ecol. 338 (1), 134–139. 10.1016/j.jembe.2006.07.003

[B61] Untergasser A. Cutcutache I. Koressaar T. Ye J. Faircloth B. C. Remm M. (2012). Primer3-new Capabilities and Interfaces. Nucleic Acids Res. 40, e115. 10.1093/nar/gks596 22730293 PMC3424584

[B62] Washington T. A. Reecy J. M. Thompson R. W. Lowe L. L. McClung J. M. Carson J. A. (2004). Lactate Dehydrogenase Expression at the Onset of Altered Loading in Rat Soleus Muscle. J. Appl. Physiol. 97 (4), 1424–1430. 10.1097/00005768-200305001-0114310.1152/japplphysiol.00222.2004 15358753

[B63] Wei J. Tian L. Wang Y. Yu L. Zhu X. (2021). Effects of Salinity, Photoperiod, and Light Spectrum on Larval Survival, Growth, and Related Enzyme Activities in the Giant Freshwater Prawn, Macrobrachium Rosenbergii. Aquaculture 530, 735794. 10.1016/j.aquaculture.2020.735794

[B64] Wen X. Chu P. Xu J. Wei X. Fu D. Wang T. (2021). Combined Effects of Low Temperature and Salinity on the Immune Response, Antioxidant Capacity and Lipid Metabolism in the Pufferfish (*Takifugu Fasciatus*). Aquaculture 531, 735866. 10.1016/j.aquaculture.2020.735866

[B65] Whittamore J. M. (2012). Osmoregulation and Epithelial Water Transport: Lessons from the Intestine of marine Teleost Fish. J. Comp. Physiol. B 182 (1), 1–39. 10.1007/s00360-011-0601-3 21735220

[B66] Wilson R. W. Wilson J. M. Grosell M. (2002). Intestinal Bicarbonate Secretion by marine Teleost Fish-Wwhy and How? Biochim. Biophys. Acta 1566 (1), 182–193. 10.1016/s0005-2736(02)00600-4 12421549

[B67] Wu L. Tang D. Shen C. Bai Y. Jiang K. Yu Q. (2021). Comparative Transcriptome Analysis of the Gills of *Cardisoma Armatum* Provides Novel Insights into the Terrestrial Adaptive Related Mechanism of Air Exposure Stress. Genomics 113 (3), 1193–1202. 10.1016/j.ygeno.2021.03.010 33711456

[B68] Yen P. T. Bart A. N. (2008). Salinity effects on reproduction of giant freshwater prawn *Macrobrachium rosenbergii* (de Man). Aquaculture 280 (1), 124–128. 10.1016/j.aquaculture.2008.04.035

[B69] Yi H. Chen X. Liu S. Han L. Liang J. Su Y. (2021). Growth, Osmoregulatory and Hypothalamic-Pituitary-Somatotropic (HPS) axis Response of the Juvenile Largemouth Bass (*Micropterus salmoides*), Reared under Different Salinities. Aquac. Rep. 20, 100727. 10.1016/j.aqrep.2021.100727

[B70] Young M. D. Wakefield M. J. Smyth G. K. Oshlack A. (2010). Gene Ontology Analysis for RNA-SEQ: Accounting for Selection Bias. Genome Biol. 11, R14. 10.1186/gb-2010-11-2-r14 20132535 PMC2872874

[B71] Yu D. F. Chen Y. Han J. M. Zhang H. Chen X. P. Zou W. J. (2008). MUC19 Expression in Human Ocular Surface and Lacrimal Gland and its Alteration in Sjögren Syndrome Patients. Exp. Eye Res. 86 (2), 403–411. 10.1016/j.exer.2007.11.013 18184611

[B72] Zhang H.-M. Liu T. Liu C.-J. Song S. Zhang X. Liu W. (2015). AnimalTFDB 2.0: a Resource for Expression, Prediction and Functional Study of Animal Transcription Factors. Nucleic Acids Res. 43 (D1), D76–D81. 10.1093/nar/gku887 25262351 PMC4384004

[B73] Zhang M. Sun Y. Liu Y. Qiao F. Chen L. Liu W.-T. (2016). Response of Gut Microbiota to Salinity Change in Two Euryhaline Aquatic Animals with Reverse Salinity Preference. Aquaculture 454, 72–80. 10.1016/j.aquaculture.2015.12.014

