# Supplementary figures and images for: Transcriptome Analysis Reveals the Molecular Response to Salinity Challenge in Larvae of the Giant Freshwater Prawn Macrobrachium rosenbergii
Source: Front Physiol. 2022 Apr 29;13:885035. doi: 10.3389/fphys.2022.885035 (PMC9099292; doi:10.3389/fphys.2022.885035)

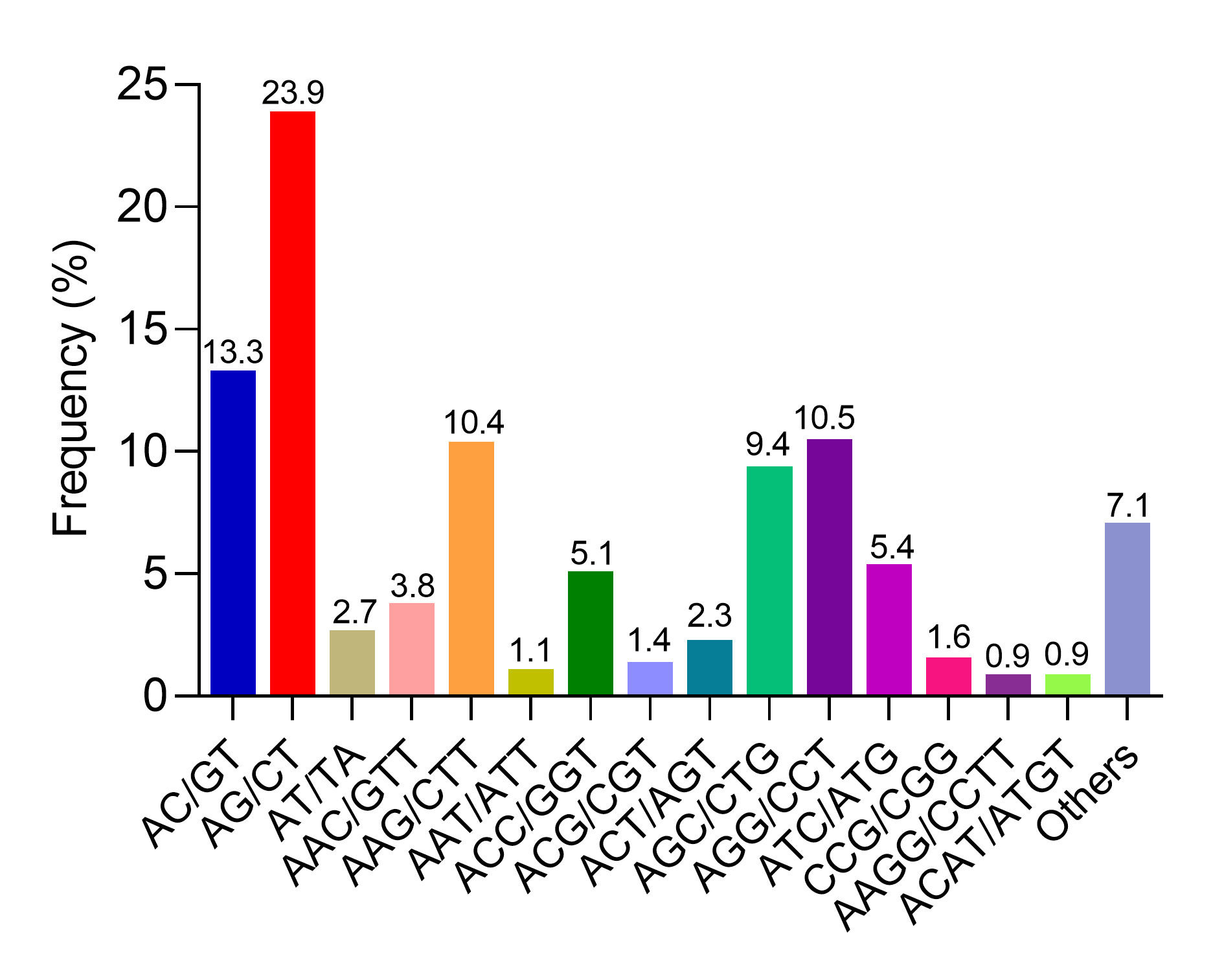

Supplement: Supplementary file 4 [file Image3.TIF]

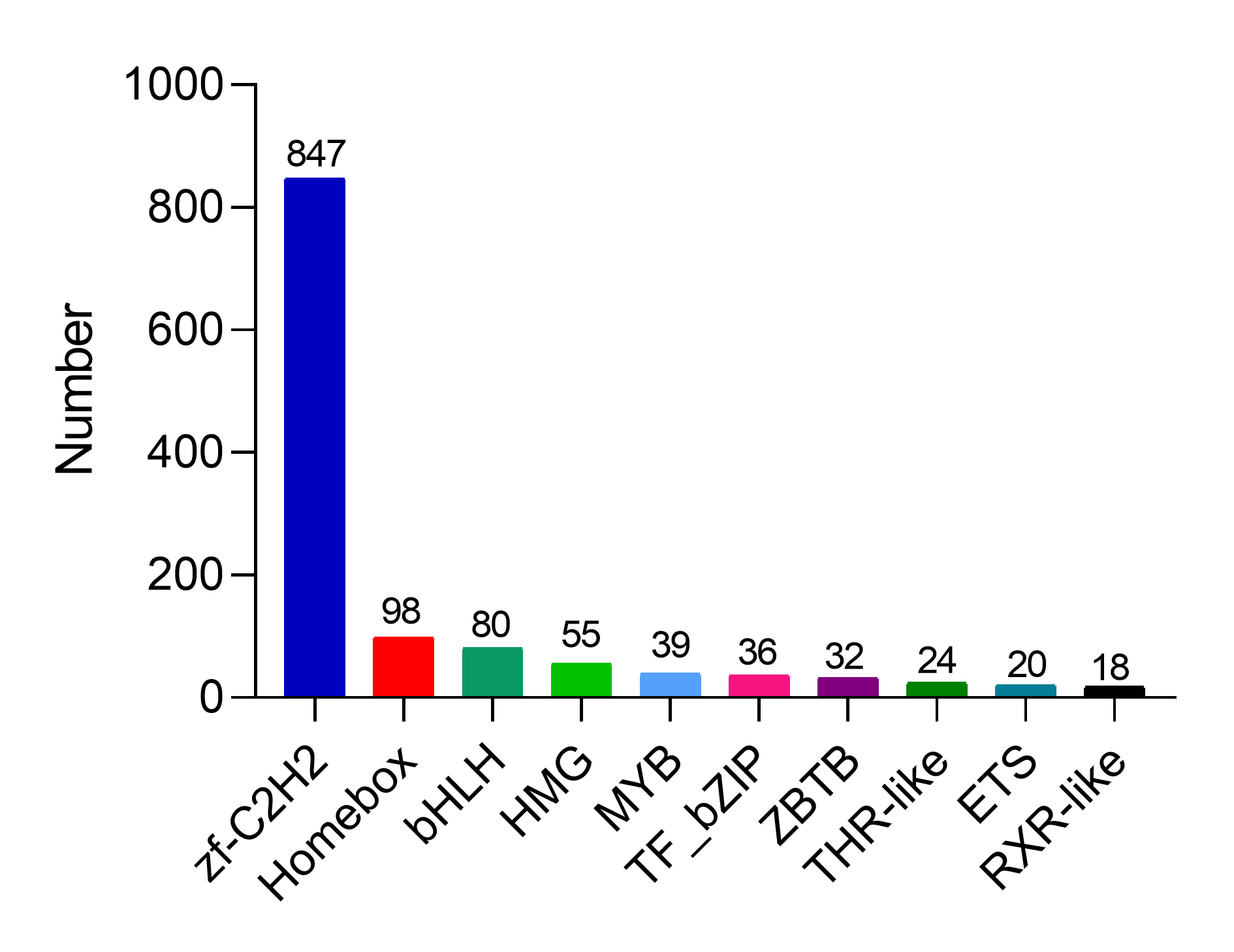

Supplement: Supplementary file 6 [file Image2.TIF]

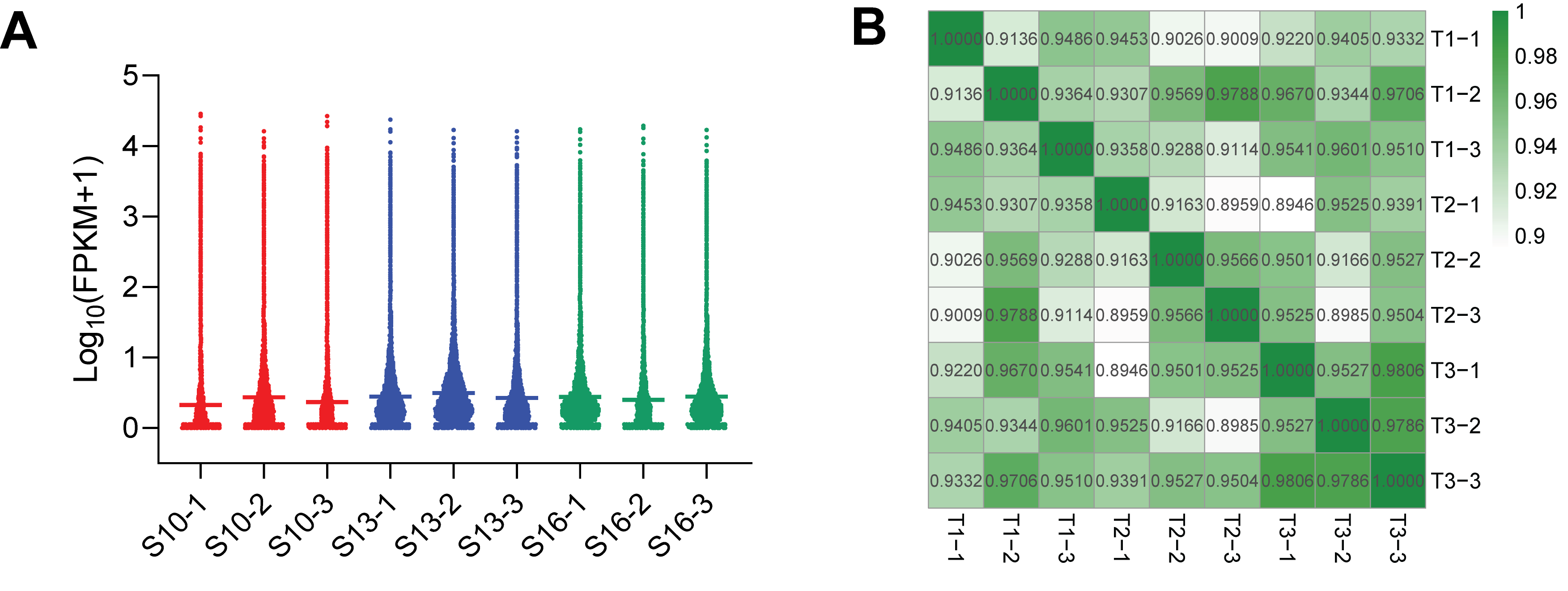

Supplement: Supplementary file 7 [file Image1.TIF]
